# Biology of Bone Sarcomas and New Therapeutic Developments

**DOI:** 10.1007/s00223-017-0372-2

**Published:** 2017-12-13

**Authors:** Hannah K. Brown, Kristina Schiavone, François Gouin, Marie-Françoise Heymann, Dominique Heymann

**Affiliations:** 1 Department of Oncology and Metabolism, Medical School, University of Sheffield, Beech Hill Road, Sheffield, S10 2RX UK; 2 European Associated Laboratory, “Sarcoma Research Unit”, Faculty of Medicine, INSERM, UMR1238, INSERM, Nantes, France; 3 Faculty of Medicine, University of Nantes, 44035 Nantes, France; 4 Institut de Cancérologie de l’Ouest, site René Gauducheau, INSERM, UMR 1232, 44805 Saint-Herblain, France; 5 European Associated Laboratory, “Sarcoma Research Unit”, INSERM, Medical School, University of Sheffield, Beech Hill Road, Sheffield, S10 2RX UK

**Keywords:** Osteosarcoma, Ewing sarcoma, Chondrosarcoma, Giant cell tumour of bone, Tumour microenvironment, Immunotherapy, Clinical trials

## Abstract

Bone sarcomas are tumours belonging to the family of mesenchymal tumours and constitute a highly heterogeneous tumour group. The three main bone sarcomas are osteosarcoma, Ewing sarcoma and chondrosarcoma each subdivided in diverse histological entities. They are clinically characterised by a relatively high morbidity and mortality, especially in children and adolescents. Although these tumours are histologically, molecularly and genetically heterogeneous, they share a common involvement of the local microenvironment in their pathogenesis. This review gives a brief overview of their specificities and summarises the main therapeutic advances in the field of bone sarcoma.

## Introduction

Bone sarcomas belong to a mesenchymal tumour family originating from bone and composed by highly heterogeneous subtypes. These tumours represent < 0.2% of malignant tumours registered in the EUROCARE database, and are considered as rare cancers and orphan tumours [1]. The three main entities are osteosarcoma, Ewing sarcoma and chondrosarcoma [2–4]. Mesenchymal stem cells (MSCs) located in most of the tissues have the ability to differentiate into various mesenchymal tissues including bone and cartilage [5]. Bone marrow and the bone environment are particularly rich in MSCs, which generate stromal cells thus supporting the haematopoiesis in addition to the bone maintenance [6, 7]. This process is in fact controlled by specific transcription factors expressed during the differentiation programme of MSCs, which orientate their differentiation towards determined cell lineages. Thus, the runx2 and sox9 master genes induce a hierarchical regulation of downstream genes modulated by MSCs and drive the differentiation of MSCs into an osteoblastic and chondroblastic lineage, respectively [4, 5]. The present review aims to give an overview on the main characteristics of bone sarcomas with a specific focus on the most recent clinical developments.

## Main Biological Characteristics of Bone Sarcomas

**Fig. 1:**
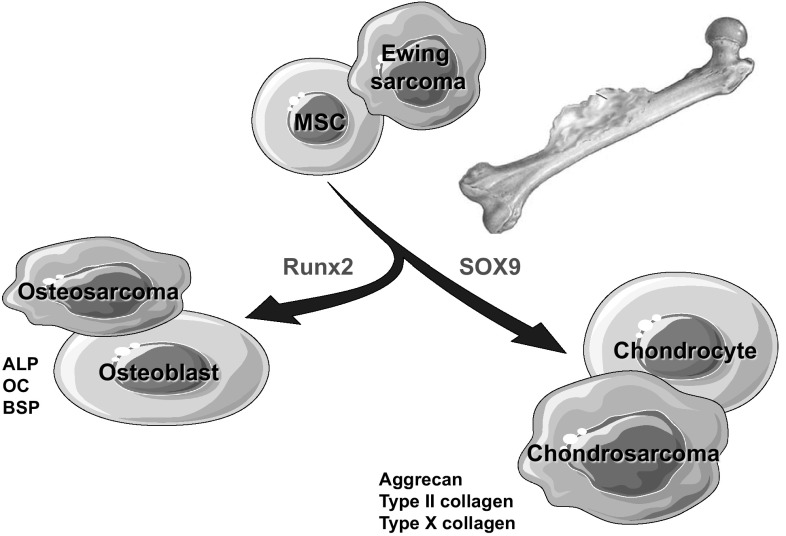
Origin of bone sarcomas. Based on the current knowledge, osteosarcoma, Ewing sarcoma and chondrosarcoma share a common mesenchymal origin. According to their differentiation level and in association with oncogenic events and an adapted microenvironment their common precursor, a “mesenchymal stem cell” could be transformed into an osteosarcoma, chondrosarcoma or an Ewing sarcoma. *Sox9* Sry-related high-mobility group box (Sox) transcription factor 9 related to chondrogenic differentiation, *Runx2* runt-related transcription factor 2 related to osteoblastogenesis, *ALP* alkaline phosphatase, *OC* osteocalcin, *BSP* bone sialoprotein

Bone sarcoma genesis can be explained by a conjunction between a minimum of one oncogenic event and an adequate microenvironment leading to the emergence of cancer, followed by its growth and potential migration to distant organs. Oncogenic events at the gene expression level (e.g. mutation, duplication, translocation) occurring during MSC differentiation increase the risk of their transformation to cancerous cells and result in the emergence of malignant osteoblastic or chondroblastic malignant cells. Indeed, osteosarcoma and chondrosarcoma cells express runx2 and sox9 in a similar manner than their non-malignant homologues [8–11]. This expression of master genes in addition to their embryologic origin and their morphology strongly establish their close relationship with MSCs (Fig. 1). In this context, osteosarcoma cells originate from MSCs that are more or less committed to the osteoblast differentiation programme in which the oncogenic events occur. Consequently, *osteosarcoma* cells can express osteoblastic markers such as alkaline phosphatase, osteocalcin or bone sialoprotein and show a strong capacity to form osteoid tissue and induce the mineralisation of extracellular matrix. *Chondrosarcoma* cells share common features with chondrocytes and express chondrocyte markers such as type II collagen or aggrecan (Fig. 1). Because chondrosarcoma cells are cytologically and phenotypically related to chondrocytes, they are able to produce cartilaginous matrix into which malignant chondrocytes become encased. Chondrosarcoma can form benign lesions in which the Hedgehog signalling pathway (such as *EXT*1 and *EXT*2 involved in the endochondral ossification) is dysregulated and evolve into malignant entities [12–14]. While osteosarcoma and chondrosarcoma can be considered as the result of a disturbed differentiation programme of MSCs, the origin of Ewing sarcoma is more controverted. Indeed, Ewing sarcoma cells are characterised by the expression of a fusion protein resulting from a chromosomal translocation between the *EWS* gene on chromosome 2 and a gene of the *ETS* family and consequently have been initially associated with the primitive neuroectodermal family of tumours [15]. However, the main frequent location of Ewing sarcoma in bone and the functional consequence of *EWS*–*FLi1* silencing in Ewing sarcoma cells fed the controversy and put a label of mesenchymal origin on Ewing sarcoma [15]. Indeed, Tirode et al. showed that the *EWS*–*FLI1* silencing in different Ewing cell lines resulted in the differentiation of sarcoma cells into mesenchymal lineages and more particularly into adipogenic and osteogenic lineages [16]. To date, its origin remains elusive with three potential hypotheses: neural crest stem cells [17], embryonic osteochondrogenic progenitor cells [18] or MSCs [16, 19]. Numerous pre-clinical models based on in vitro approaches and in vivo investigations (e.g. rat, mouse, zebrafish) mimicking the human disease have been proposed and are currently used to study the pathogenesis of bone sarcomas and/or for screening new drugs [20–28].

## Main Clinical Characteristics of Bone Sarcomas

Osteosarcoma, Ewing sarcoma and chondrosarcoma are separated into three different clinical entities identifiable by the patient populations affected, their localisation and their biological characteristics (Table 1). Osteosarcoma is the most frequent malignant primary bone tumour with a higher incidence in adolescent and young adults. Two peaks of incidence are conventionally described: (i) a main peak at 18 years and (ii) a second peak at 60 years with poor prognosis corresponding frequently to secondary osteosarcoma developed after radiotherapy or after Paget disease of bone [2, 3]. All osteosarcomas are characterised by the presence of a mineralised osteoid matrix produced by cancer cells and which results in the typical radiographic appearances called “sunburst” pattern [4, 29]. Osteosarcoma are very heterogeneous tumours (intra- and inter-tumoural heterogeneity) as revealed by the multiple histological subtypes according to the degree of cancer cell differentiation and consequently the quality of the extracellular matrix secreted (e.g. osteoblastic, chondroblastic, fibroblastic, telangiectatic osteosarcoma). The main affected areas of osteosarcoma are the metaphysis of the long bones with a preference to the proximal end of the tibia/fibula corresponding to the location of the growth plate. Genetic analyses confirmed the high heterogeneity of osteosarcoma [30–32]. Bousquet et al. identified for instance more than 80 point mutations and some deletions related to more than 80 genes [30]. Kovac et al. interestingly identified a BRCAness signature in osteosarcoma which could be exploited as a new therapeutic targeting [31]. The overall survival of osteosarcoma patients is very dependent on their metastatic status at the time of diagnosis with a survival rate for patients with localised disease of around 65% after 5 years; however, when lung metastases are detected, survival drops to 30% (Table 1). Around 10–20% of patients show clinically detectable metastases at time of diagnosis and 85–90% are located in the lungs.

**Table 1:** Characteristics of the three main bone sarcomas

Tumour type	Ratio male/female	Frequency^a^	Peak of incidence (years)	Principal localisations	Survival rate
Osteosarcoma	1.4	0.2–0.3/100,000/year (general population) 08–1.1/100,000/year at age 15–19	Main peak: 18 Secondary peak: 60	Metaphysis of long bones Distal end of femur + proximal end tibia/fibula (60%)	60–70% after 5 years 30% after 5 years (with lung metastases)
Ewing sarcoma	1.5	0.3/100,000/year	15	Flat bones (60%) Metaphysis of long bones (40%) and soft tissues	66% at 5 years and 20% at 5 years for poor responders
Chondrosarcoma	1	0.2/100,000/year	45	Pelvic bone, femur, proximal humerus, scapula	50–60% at 10 years according the histological grade

^a^Source: ref [2]

Ewing sarcoma is the second main represented bone sarcoma with 0.3/100,000/year. This bone sarcoma subtype accounts for 2% of childhood cancers, is more predominant in male than female with a sex male/female ratio around 1.5 and has a peak of incidence at 15 years. Sixty percent of Ewing sarcomas develop in flat bones and 40% affect the metaphysis of long bones (Table 1). Similar to osteosarcoma, the overall survival is also associated with the metastatic status of patients. For localised tumours, the overall survival is 50–60% at 5 years, which drops to only around 20% for metastatic sarcoma. At time of diagnosis, 20–25% of patients show clinically detectable metastases [33–35]. Although Ewing sarcoma is the most homogeneous entity among bone sarcomas, composed of undifferentiated round cancer cells characterised by CD99-, FLI1-, HNK1- and CAV1-positive immunostaining associated with limited stromal components [36], recent work demonstrated in contrast their heterogeneity [37–40]. Previous studies highlighted only a few recurrent somatic mutations in Ewing sarcomas (*TP53, STAG2, CDKN2*) [38, 41, 42]. However, more recent studies by Zhang et al. used next-generation sequencing (Ion AmpliSeq™ Cancer Hotspot Panel v2) to identify a series of five new mutations (*KDR, STK11, MLH1, KRAS* and *PTPN11*) related to a higher proliferation index and revealing a higher tumour heterogeneity than initially suspected [37]. This heterogeneity is not restricted to the genetic patterns but can be extended to epigenetic profiles [39]. Indeed, Sheffield et al. showed heterogeneous DNA methylation profiles between different tumours, which could reflect a continuum between mesenchymal and stem cell signatures in link with the EWS–FLI1 signature [39]. In addition, the expression levels of EWS–FLi1, which are variable in a tumour tissue, have a functional impact on cell migration. EWSR1–FLi1^high^ cells are characterised by high proliferation activity, while EWSR1–FLi1^low^ have a marked propensity to migrate, invade and metastasise [40].

Chondrosarcoma is the third entity of bone sarcoma in term of incidence with around 0.2 new cases per 100,000 each year and similar incidence between male and female (Table 1). Similar to all bone sarcomas, several subtypes can be identified according to their histological characteristics [43–46] and are classified as low, intermediate or high grade on the basis of histopathological features [47]. Chondrosarcomas are characterised by a tumour chondrocyte-derived hyaline-like extracellular matrix, which eventually encases the cancer cells. The tumour tissue is organised in a mosaic of lobules separated by fibrous tissue. In addition, chondrosarcomas exhibit low vascularisation in contrast to osteosarcoma and Ewing sarcomas. Heterogeneity is also a hallmark of chondrosarcomas, which are associated with a complex cytogenetic signature [48, 49]. Thus, somatic mutations in isocitrate dehydrogenase (IDH)-1 or -2 are frequent (around 56%) in central and periosteal cartilaginous tumours and absent in endochondroma [50]. In addition to mutations in *IDH1, IDH2, EXT (*exostosin) and more conventional genes associated with cancer progression such as *TP53* or *Rb1*, Tarpey et al. identified COL2A1 mutations (insertions, deletions and rearrangements) in the third cases [51]. The principal localisations of chondrosarcomas are pelvic bone, scapula and long bones (Table 1). While high-grade chondrosarcomas can be associated with metastases, these tumours are characterised by a high rate of local recurrence and consequently by a high morbidity [52, 53]. Osteosarcoma, Ewing sarcoma and chondrosarcoma are then characterised by a marked heterogeneity at the histological, genetic and epigenetic levels.

## Etiology of Bone Sarcomas: The Microenvironment as the Driver of Cancer Progression

In addition to c-fos which has been associated with osteosarcoma formation due to its contribution in osteoblast differentiation [54, 55], some genetic predispositions have been linked with osteosarcoma development in hereditary syndromes such as Li-Fraumeni (p53 mutation) [56], Rothmund-Thompson [57], Werner [58] or Bloom syndromes (mutations of helicase genes) [59, 60], or retinoblastoma familial cancers [61]. Hereditary multiple exostoses (familial osteochondromatosis or diaphyseal aclasis) is an inherited genetic disease associated with osteochondromas and with *EXT1* and *EXT2* mutations [14, 62]. Even if several studies evaluated the risk of malignant transformation of multiple exostoses, the most recent study identified this risk at relatively low level (2.7%) with the development of low-grade chondrosarcomas [63]. However in most of the cases, patients do not show any predisposition genes and bone sarcomas are sporadic cases which could be explained by a close relationship with their local microenvironment altered during the malignant transformation process [64–68]. The “seed and soil” theory proposed by Stephen Paget at the end of the nineteenth century gives a partial explanation of bone sarcoma formation [69]. At the early stage of the disease, proliferation of bone sarcoma cells in the bone environment leads to the dysregulation of the balance between osteoblasts and osteoclasts, in favour of an exacerbated osteoclast differentiation and local bone resorption. In turn, resorptive osteoclasts release pro-tumoral factors (e.g. cytokines, extracellular matrix components) initially trapped into the organic matrix of bone tissue [70]. The demonstration of this vicious cycle between osteoclasts and bone sarcoma cells has stimulated numerous pre-clinical and clinical investigations that revealed the decrease of tumour bone sarcomas after targeting of osteoclasts using anti-resorptive agents [71–75]. In addition to their anti-resorptive activities, nitrogen-containing bisphosphonates could have a direct anti-proliferative activity on cancer cells [76, 77]. On the contrary, Endo-Munoz et al. showed the deleterious effect of osteoclastogenesis inhibition after zoledronic acid treatment which was associated with an increase of lung metastases in an osteosarcoma model [78]. The role of osteoclasts in bone sarcoma development is still unclear and osteoclasts could act as a pro-tumoral factor in the early stage of the disease due to their pro-angiogenic activity [79] and could exert the opposite role at a later stage of the disease [80].

Bone sarcoma development could be explained by the conjunction of multiple factors: (i) one or more oncogenic events from which the malignant transformation is initiated. The risks of genetic aberrations at the gene expression level (e.g. mutation, deletion, amplification) could increase with the proliferation rate of the cells of interest such as MSCs/osteoblasts during bone growth. A first mutation could lead to a chromosomal instability and consequently to the appearance of new oncogenic events [31]. (ii) A favourable microenvironment is a prerequisite for the growth of cancer cells. The differential repartition of bone sarcomas according to their subtypes are in favour of this theory. Furthermore, numerous studies demonstrated that MSCs induce pro-proliferative effects on bone sarcoma and promote osteosarcoma stemness strengthening the “seed and soil” theory [81, 82]. Local acidosis derived from the tumour growth and tumour-associated osteolysis has in return a strong impact on the stemness of MSCs [83, 84]. The bilateral dialogue established between cancer cells and their neighbours is a central aspect of bone sarcoma development. The diverse modes of communication include soluble factors (e.g. chemokines, cytokines), direct cell interactions and extracellular vesicles [64–66]. Gap junctions are intercellular channels composed of transmembrane proteins named connexons that allow direct intercellular communication between two adjacent cells. Recent data investigated at the single-cell level showed intercellular communications through gap junctions between osteosarcoma cells and various other cell types [85]. Functional gap junctions have been observed between osteosarcoma cells and MSCs depending on their differentiation levels, and between cancer cells and endothelial cells. In contrast, while all bone cells express gap junctions, no gap junction-dependent communication has been demonstrated with macrophages, osteoclasts or osteocytes [86–88]. Gap junctions are clearly involved in the tumour development and the loss of connexin43 expression in Ewing sarcoma cells favours the development of the primary tumour growth [89]. Another way of cell communication is transfer of extracellular vesicles loaded with proteins, mRNA and microRNA. Thus, it has been suggested that osteosarcoma cells are able to resist the effects of chemotherapeutic treatment such as doxorubicin by transferring exosomes carrying specific multidrug resistance factors (e.g. MDR-1, Pgp) from resistant to non-resistant cancer cells [90]. Recently, Baglio et al. described the education of MSCs by tumour-secreted extracellular vesicles [91]. These authors demonstrated the ability of osteosarcoma cells to incorporate TGF-β into extracellular vesicles which induced production of IL-6 in MSCs. IL-6 is in turn associated with an increase of tumour growth [92]. A vicious cycle is then established between MSCs and sarcoma cells through the release of extracellular vesicles.

**Fig. 2:**
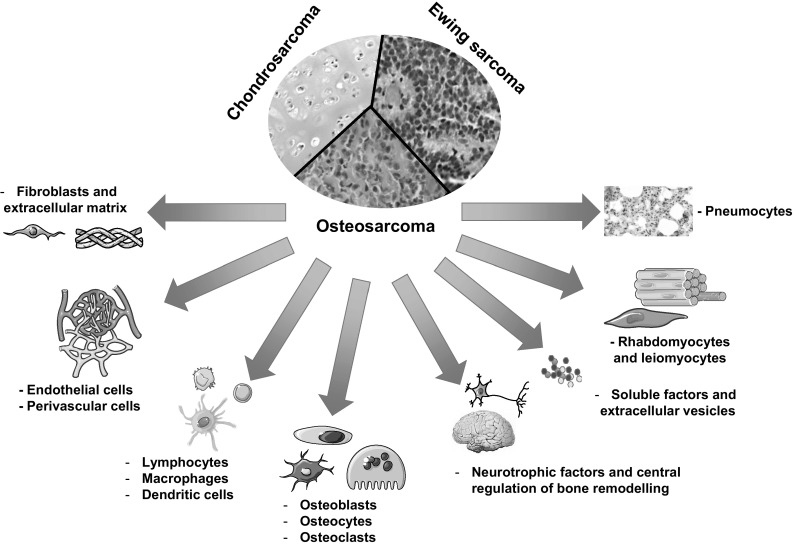
The tumour microenvironment contributes to the control of bone sarcoma formation, their recurrence and associated metastatic process. The bone sarcoma microenvironment is composed of highly diversified cell populations forming specific local niches: vascular niche, immune niche, bone niche, muscular and pulmonary niches (e.g. metastatic niches), neuronal control and activity of neurotrophic factors. These various cell types establish a mutual dialogue with sarcoma cells through physical contact, the release of soluble factors or the formation of extracellular vesicles. All these communications will lead to strong alterations of the microenvironment (e.g. qualitative modifications of the extracellular matrix) and the behaviour of cancer cells, which increase their proliferation, and/or invasion/migration properties

The bone sarcoma microenvironment is not restricted to MSCs but is a very complex and dynamic environment (Fig. 2). This environment can be described as “niches” including bone, vascular and immune niches and more specific niches such as muscles and lung parenchyma for invading and metastatic cells. Even though there is no evidence of the correlation between the vessel density and the metastatic process in bone sarcomas, endothelial cells are strongly involved in the intra/extravasation of cancer cells. Recently, new regulators including brain, neuronal network and neurotrophic factors should be added to the list. It is now well recognised that the brain can act as a master regulator of bone mass [93, 94]. Bone remodelling is indeed regulated by a rich innervation, which is the source of neurotrophic factors, hormones and neurotransmitters [95]. Released locally or into the blood stream, these soluble factors could target bone sarcoma cells [96, 97]. The most recent evidence has been given by Punzo et al. who showed the anti-proliferative, pro-apoptotic and anti-invasive effects of endocannabinoid and endovanilloid systems in osteosarcoma [98] (Fig. 2). The bone environment is relatively specific to bone sarcomas and bone cells have been suspected to contribute to their development. Indeed, as described above, the blockade of bone resorption by bisphosphonates inhibits the tumour growth in pre-clinical models of osteosarcoma [92] and Ewing sarcoma [71] and slows down recurrent tumour progression after intralesional curettage in chondrosarcoma [76, 99]. Unfortunately, the results of a phase III clinical trial associating conventional chemotherapy and bisphosphonate (zoledronate) do not recommend this therapeutic strategy in osteosarcoma [100]. The lack of significant efficacy can be explained by the disparity of bisphosphonate or RANKL-blocking antibody efficacy observed using the parameters of bone remodelling in different mouse strains [101]. Alternatively, bisphosphonates could modulate macrophage differentiation through complex mechanisms. Tumour-associated macrophages (TAMs) can be subdivided in two types of populations, M1-polarised macrophages considered as antitumour effectors and M2-polarised macrophages, which are defined as pro-tumour modulators due to their positive impact on the neoangiogenic process [102]. In breast cancer models, it has been shown that cancer cells secrete soluble factors modulating macrophages towards the M2 state. Zoledronate counteracts this differentiation and favours a cytotoxic immune response linked with the differentiation of TAMs towards the M1 subtype [103]. In mesothelioma, zoledronate impairs the polarisation of TAMs to the M2 phenotype but leads to the accumulation of immature myeloid cells, which could reduce its effects [104]. In bone sarcoma, TAMs also appeared as key effectors of the pathogenesis [105–107]. Indeed, the macrophage infiltration in osteosarcoma is correlated with metastatic suppression [105] and osteosarcoma cells dysregulate the balance of M1/M2 macrophages [106]. An abundant M2 macrophage infiltrate is consequently in favour of a metastatic profile [106]. In Ewing sarcoma, the targeting of TAMs by liposome-encapsulated clodronate that inhibits simultaneously M1 and M2 macrophages leads to a decrease of tumour growth [107]. Overall, these results demonstrate the key role of macrophages which regulate the development of bone sarcoma according to their number and M1/M2 phenotype. The role of the immune niche in bone sarcoma development is not restricted to TAMs and is also controlled by dendritic cells, tumour infiltrating lymphocytes or mast cells [108–110].

## Recent Therapeutic Developments

**Fig. 3:**
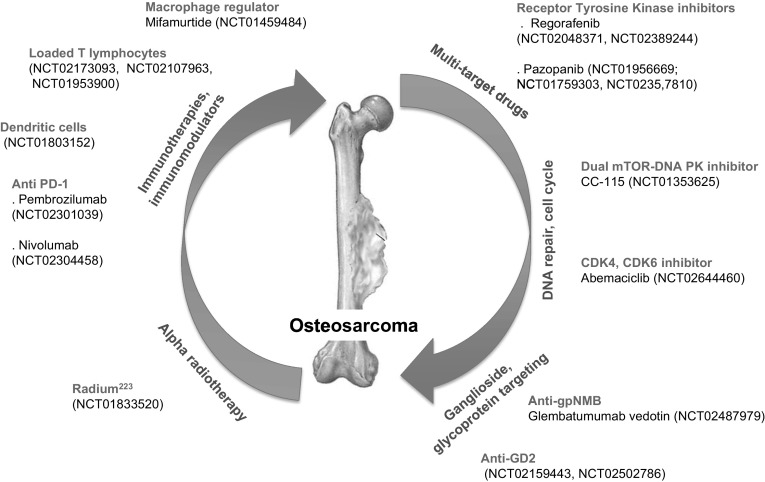
Recent on-going clinical trials in osteosarcoma. Numerous therapeutic approaches are in clinical development and are based on specific and direct targeting of cancer cells (e.g. DNA repair, cell cycle or glycoprotein targeting), or indirect targeting of cancer cells by modulation of their microenvironment (e.g. immunotherapies). After integration in extracellular tumour bone matrix, alpha radiotherapeutic agents can indirectly kill the cancer cells. NCT: National Clinical Trial NuClinicalTrials.gov registry Number

Although current conventional treatments are relatively similar for osteosarcoma and Ewing sarcoma combining chemotherapy and surgery, the mainstay of local tumour control in chondrosarcoma is surgery with adequate margins (margins of normal tissue). Indeed, chemotherapy and radiotherapy are ineffective in the treatment of local and advanced chondrosarcoma patients. Consequently, both therapeutic approaches have limited impact in the management of these patients [111]. Unfortunately, adequate margins can only be achieved in 45–75% of patients. Inadequate margins are related to a high risk of local recurrence. Recent work validated the cryosurgery after intralesional curettage for low-grade chondrosarcoma. The technique appears safe and effective in selected patients [111]. Chemotherapy is recommended for high-risk chondrosarcoma and dedifferentiated chondrosarcoma but there is no recognised consensus defining the protocol and time schedule. The conventional therapeutic approach to osteosarcoma and Ewing sarcoma combines surgery (preoperative or neoadjuvant) and after chemotherapy (postoperative or adjuvant) and long-term (6–12 months) polychemotherapy [112–115]. The conventional cocktail used in osteosarcoma is composed by a minimum of three drugs (reference combination: doxorubicin, cisplatin, methotrexate). Ifosfamide is the fourth drug used in osteosarcoma. Radiotherapy can be used when adequate surgery is impossible and for high-risk locations (e.g. spine); however, osteosarcomas are usually considered as radioresistant. In Ewing sarcoma, chemotherapy includes vincristine, ifosfamide, doxorubicin and etoposide. In addition, patients will receive radiotherapy since Ewing sarcoma responds relatively well to irradiation [116]. Several on-going clinical trials are studying new regimens of high doses of chemotherapy in combination with radiotherapy (Table 2). However, most conventional chemotherapy commonly results in relatively poor therapeutic responses, which has led to the development of new compounds with new therapeutic targets (Tables 2, 3; Fig. 3).

**Table 2:** Recent drug development in Ewing sarcoma

Drug	Reference	Title	Phase	Experimental plan	Primary outcome	Patients	Status
Temozolomide Irinotecan Vincristine Adriamycin Ifosfamide Etoposide Cyclofosfamide Busulfan Melfalan Celecoxib	NCT02727387	Study with high doses of chemotherapy, radiotherapy and consolidation therapy With cyclofosfamide and anti-cyclooxygenase 2, for the metastatic Ewing sarcoma	II	Two cycles of temozolomide (500 mg/m^2^) + irinotecan (250 mg/m^2^) and two cycles of vincristine (1.4 mg/m^2^) + adriamycin (90 mg/m^2^) + Ifosfamide (9 g/m^2^) alternes with two cycles of cyclofosfamide (4 g/m^2^) + etoposide (600 mg/m^2^) followed by radiotherapy (42–54 Gy) and two cycles of Ifosfamide (9 g/m^2^) + etoposide (300 mg/m^2^) alternes with to two cycles of vincristine (1.4 mg/m^2^) + adriamycin (80 mg/m^2^) + cyclofosfamide (1.2 g/m^2^) and busulfan (0.8–1.2 mg/kg) + melfalan (140 mg/m^2^) + PBSCT and 6 months with celecoxib cyclofosfamide	Overall survival Event-free survival	70	Recruiting End: 2020
Cyclophosphamide Doxorubicin Vincristine Ifosfamide Etoposide Temozolomide Irinotecan	NCT01864109	A phase II trial of irinotecan and temozolomide in combination with existing high dose alkylator based chemotherapy for treatment of patients with newly diagnosed Ewing sarcoma	II	Patients with localised disease: *six cycles of the combination* as “maintenance” therapy following standard chemotherapy *Cycles 4–6* including Ifosfamide 2800 mg/m^2^/day on days 1–5 Etoposide 100 mg/m^2^/day on days 1–5 *Cycle 7* including: Cyclophosphamide on days 1 and 2 at a dose of 2100 mg/m^2^/day, or for patients < 10 years of age at a dose of 70 mg/kg/day Doxorubicin on days 1 and 2 at a dose of 37.5 mg/m^2^/day Vincristine on day 1 at a dose of 2 mg/m^2^ or 0.067 mg/kg (whichever is lower, to a max dose of 2 mg) *Cycles 8–13* including: Irinotecan i.v. on 10 days over weeks 1 and 2 of a cycle at a dose of 20 mg/m^2^/day Temozolomide daily on the first 5 days of irinotecan administration at a dose of 100 mg/m^2^/day p.o. or i.v. Patients with metastatic disease: ten cycles of the combination intercalated between the final *4 cycles of standard chemotherapy* *Cycles 4, 5, 7, 8, 10, 11, 13, 14, 16 and 17*: Irinotecan i.v. on 10 days over weeks 1 and 2 of a cycle at a dose of 20 mg/m^2^/day Temozolomide daily on the first 5 days of irinotecan 100 mg/m^2^/day p.o. or i.v. *Cycles 6, 9 and 12* Ifosfamide 2800 mg/m^2^/day on days 1–5 Etoposide 100 mg/m^2^/day on days 1–5 *Cycle 15*: Cyclophosphamide on days 1 and 2 at a dose of 2100 mg/m^2^/day, or for patients < 10 years of age at a dose of 70 mg/kg/day Doxorubicin on days 1 and 2 at a dose of 37.5 mg/m^2^/day Vincristine will be given on day 1 at a dose of 2 mg/m^2^ or 0.067 mg/kg (whichever is lower, to a max dose of 2 mg)	Event-free survival of patients with localised disease Progressive disease according to RECIST 1.1	83	Recruiting End: 2019
Zoledronic acid Buslphan Treosulfan	NCT00987636	Study in localized and disseminated Ewing sarcoma (EWING2008)	III	Zoledronic acid i.v. at 28-day intervals beginning with cycle 6 of VAC/VAI consolidation chemotherapy for a total period of 9 months *Patients < 18 years*: 0.05 mg/kg by i.v. infusion 30 min^− 1^ h *Patients > 18 years* will receive a bodyweight-dependent dose: Patients > 40 kg receive 4 mg by i.v. infusion 30 min–1 h. Patients 20–40 kg: 2 mg by i.v. infusion 30 min to 1 h	Improvement of event-free survival compared to the absence of bisphosphonate	1163	Recruiting End: 2019
Olaparib (PARP inhibitor) Temozolomide Irinotecan	NCT01858168	Phase I Study of olaparib and temozolomide in adult patients with recurrent/metastatic Ewing sarcoma following failure of prior chemotherapy	I	Arm 1: olaparib, p.o. twice per day on days 1–7 (week 1) of each cycle Temozolomide, p.o. once per day on days 1–7 (week 1) of each cycle irinotecan, given by i.v. once per day on days 1–7 of each cycle Arm 2: olaparib p.o. twice per day on days 1–7 (week 1) of each cycle Temozolomide, p.o. once per day on days 1–7 (week 1) of each cycle	Maximum tolerated dose	93	Recruiting End: 2019
Niraparib (PARP inhibitor) Irinotecan Temozolomide	NCT02044120	ESP1/SARC025 global collaboration: a phase I study of a combination of the PARP inhibitor, niraparib and temozolomide or irinotecan in patients with previously treated, incurable Ewing sarcoma	I	Up to 12 cycles of niraparib and temozolomide (Arm 1) or niraparib and irinotecan (Arm 2)	Maximum tolerated dose Dose-limiting toxicity	50	Recruiting End: 2019
Pbi-shRNA™ EWS/FLI1 Type 1 LPX	NCT02736565	Phase I trial of Pbi-shRNA™ EWS/FLI1 type 1 lipoplex (LPX) in subjects with advanced Ewing sarcoma	I	Escalation cohorts up to a dose of 0.156 mg/kg of DNA/single dose (i.v. twice a week for 4 weeks for a total of eight infusions of the product per cycle followed by 2 weeks of rest)	Safety	22	Recruiting End: 2018
TK216 Inhibitor of protein–protein interactions of EWS–FLI1 fusion protein	NCT02657005	A phase 1, dose escalation study of intravenous TK216 in patients with relapsed or refractory Ewing sarcoma	I	Dose escalation	Maximum tolerated dose Determination of the dose-limiting toxicity	45	Recruiting End: 2018
Temozolomide Irinotecan Vigil	NCT02511132	A two-part phase IIb trial of Vigil (Bi-shRNAfurin and GMCSF augmented autologous tumor Cell Immunotherapy) in Ewing’s sarcoma	IIb	Temozolomide p.o. 100 mg/m^2^ daily (days 1–5, total dose 500 mg/m^2^/cycle) Irinotecan p.o. 50 mg/m^2^ daily (days 1–5, total dose 250 mg/m^2^/cycle), or irinotecan i.v. 20 mg/m^2^ daily (days 1–5, total dose 100 mg/m^2^/cycle) Peg-filgrastim 100 μg/kg (day 6) subcutaneously Vigil 1.0 × 10^7^ cells/injection, intradermally on day 15 and every 43 weeks thereafter. One cycle = 21 days	Safety profile of Vigil immunotherapy IFNγ ELISPOT conversion rate of subjects treated with Vigil immunotherapy	9	Recruiting End: 2018
Cyclophosphamide Doxorubicin Hydrochloride Etoposide Ganitumab Ifosfamide Vincristine Sulfate	NCT02306161	Combination chemotherapy with or without Ganitumab in treating Patients with newly diagnosed metastatic Ewing sarcoma	III		Time to adverse analytic event (EFS), defined to be disease-related event, diagnosis of a second malignant neoplasm or death	330	
Liposomal Doxorubicin	NCT02557854	HIFU hyperthermia with liposomal doxorubicin (DOXIL) for relapsed or refractory pediatric and young adult solid tumors	I	Liposomal doxorubicin (Doxil) 50 mg i.v. every 4 weeks followed by magnetic resonance high-intensity focused ultrasound hyperthermia (MR-HIFU) for 30 min every 4 weeks	Rate of dose-limiting toxicities	14	Recruiting End: 2019
Irinotecan sucrosofate liposomes Cycophosphamide	NCT02013336	Phase 1 study of MM-398 plus cyclophosphamide in pediatric solid tumors	I		Maximum tolerated dose	30	Recruiting End: 2017
Regorafenib (tyrosine kinase inhibitor)	NCT02048371	SARC024: a blanket protocol to study oral regorafenib in patients with refractory liposarcoma, osteogenic sarcoma, and Ewing sarcomas	II	Regorafenib 160 mg daily; 21 days on and 7 days off	Progression-free survival	126	Recruiting End: 2019
Cabozantinib (tyrosine kinase inhibitor)	NCT02867592	Phase 2 trial of XL184 (Cabozantinib) an oral small-molecule inhibitor of multiple kinases, in children and young adults with refractory sarcomas, Wilms tumor, and other rare tumors	II	Cabozantinib p.o.	Objective response assessed by RECIST1.1	110	Recruiting End: 2018
Entrectinib (tyrosine kinase inhibitor)	NCT02650401	Study of RXDX-101 in children with recurrent or refractory solid tumors and primary CNS tumors, with or without TRK, ROS1 or ALK fusions	I	Escalating doses Entrectinib p.o.	Maximum tolerated dose	190	Recruiting End: 2019
Erlotinib (EGFR inhibitor) Temozolomide	NCT02689336	Erlotinib in combination with temozolomide in treating relapsed/recurrent/refractory pediatric solid tumors	II	Erlotinib p.o., 85 mg/m^2^/dose once a day continuously (every day of a 28-day cycle) Temozolomide p.o. 180 mg/m^2^/dose once a day on days 1–5 of a 28-day cycle	Overall response rate	30	Recruiting End: 2020
Enoblituzumab (B7-H3 antibody)	NCT02982941	Enoblituzumab (MGA271) in children with B7-H3-expressing Solid tumors	I	Enoblituzumab i.v. on a weekly schedule for up to 96 doses (approximately 2 years) in children and young adults with B7-H3-expressing relapsed or refractory malignant solid tumours	Safety Tolerability PK, PD Immunogenicity Preliminary antitumour activity	112	Recruiting End: 2022
Nivolumab (PD1 inhibitor) Ipilimumab (anti-CTLA4 antibody)	NCT02304458	Nivolumab With or Without Ipilimumab in Treating Younger Patients With Recurrent or Refractory Solid Tumors or Sarcomas	I–II	Nivolumab i.v. Ipilimumab i.v.	Maximum tolerated dose of nivolumab Response rate of nivolumab combined with ipilimumab according to RECIST	352	Recruiting End: 2020
Abemaciclib (CD4–CDK6 inhibitors)	NCT02644460	Abemaciclib in children with DIPG or recurrent/refractory solid tumors (AflacST1501)	I	Escalating doses Abemaciclib (LY2835219) p.o. on a twice daily basis continuously for 28 days, which defines one cycle	Maximum tolerated dose	50	Recruiting End: 2020
TB-403 (anti-PLGF monoclonal antibody)	NCT02748135	A two-part study of TB-403 in pediatric subjects with relapsed or refractory medulloblastoma	I–II	Drug: TB-403 20 mg/kg Drug: TB-403 50 mg/kg Drug: TB-403 100 mg/kg Drug: TB-403 ≤ 175 mg/kg	Maximum tolerated dose	36	Recruiting End: 2018
Expanded NK cells	NCT02409576	Pilot study of expanded, activated haploidentical natural killer cell infusions for sarcomas (NKEXPSARC)	I–II		Disease response after expanded activated NK cell infusion	20	Recruiting End: 2018
hu14.18K322A Human anti GD2 antibody	NCT02159443	Pretreatment anti-therapeutic antibodies (PATA) in patients treated with hu14.18K322A Antibody	Obs.		To determine whether pretreatment anti-therapeutic antibodies (PATA) represent antibodies reactive against an epitope (allotypic determinant) found on the anti-GD2 antibody hu14.18K322A	100	Recruiting End 2019

*Obs*. observational

**Table 3:** Recent drug development in chondrosarcoma

Drug	Reference	Title	Phase	Doses	Primary outcome	Patients	Status
Regorafenib (tyrosine kinase inhibitor)	NCT02389244	A phase II study evaluating efficacy and safety of regorafenib in patients with metastatic bone sarcomas	II	160 mg/d once daily for the 3 weeks on/1 week off plus Best Supportive Care (BSC) until progression (according to RECIST 1.1)	Progression-free survival defined using RECIST 1.1	132	2014–2020
Pazopanib (tyrosine kinase inhibitor)	NCT01330966	Study of pazopanib in the treatment of surgically unresectable or metastatic chondrosarcoma	II	800 mg p.o. once daily for 28 days	Disease control at week 16	47	2011–2017
Pazopanib	NCT02066285	Trial of pazopanib in patients with solitary fibrous tumor and extraskeletal myxoid chondrosarcoma	II	800 mg (2 × 400 mg or 4 × 200 mg) as a single agent once daily continuously	Therapeutic response rate measured using Choi and RECIST 1.1 criteria	70	2014–2018
Gemcitabine + pazopanib	NCT01532687	Gemcitabine hydrochloride with or without pazopanib hydrochloride in treating patients with refractory soft tissue sarcoma	II	Gemcitabine hydrochloride i.v. on days 1 and 8 and pazopanib hydrochloride p.o. on days 1–21. Courses repeat every 21 days in the absence of disease progression or unacceptable toxicity	Progression-free survival	80	2012–2018
Imatinib (tyrosine kinase inhibitor)	NCT00928525	Imatinib in patients with desmoid tumor and chondrosarcoma	II	800 mg p.o./day (400 mg b.i.d.) for a maximum of 24 months	Tumour response by imaging techniques	35	2009–2016
Dasatinib (tyrosine kinase inhibitor)	NCT00464620	Trial of dasatinib in advanced sarcomas	II	70 mg of Dasatinib p.o., twice daily, for 28-day cycles	Response rate and the 6-month progression-free survival rates	386	2007–2017
Vismodegib (Hedgehog inhibitor)	NCT01267955	Vismodegib in treating patients with advanced chondrosarcomas	II	Vismodegib p.o. on days 1–28. Courses repeat every 28 days in the absence of disease progression or unacceptable toxicity	Objective therapeutic response rate measured using RECIST 1.1 criteria	45	2010–2016
Linsitinib (inhibitor of IGF1-R)	NCT01560260	Linsitinib in treating patients with gastrointestinal stromal tumors	II	Oral linsitinib 150 mg B.I.D. on days 1–28. Courses repeat every 28 days in the absence of disease progression or unacceptable toxicity	Therapeutic response evaluated according to RECIST 1.1	20 including GIST and paraganglioma	2012–2016
Tazemetostat (EZH2 inhibitor)	NCT02601950	A phase 2 study of the EZH2 inhibitor tazemetostat in pediatric subjects with relapsed or refractory INI1-negative tumors or synovial sarcoma	I	Tazemetostat p.o. 800 mg B.I.D. administered in continuous 28-day cycles	Objective response, progression-free survival	180 (including INI1-negative tumours or any solid tumour with an EZH2 gain of function mutation)	2015–2017
Metformin + chloroquine	NCT02496741	Metformin and chloroquine in IDH1/2-mutated solid tumors (MACIST)	Ib	Metformin administered in a 3 + 3 dose-escalation schedule and chloroquine administered in a fixed dose	Maximum tolerated dose of metformin + chloroquine	20	2015–2016
Sirolimus (mTOR inhibitor) + cyclophosphamide	NCT02821507	Sirolimus and cyclophosphamide in metastatic or unresectable myxoid liposarcoma and chondrosarcoma	II	Sirolimus 4 mg p.o. daily and cyclophosphamide p.o. 200 mg day 1–7 and 15–21 in a 4-week schedule	Growth modulation index until disease progression (time frame: 16 weeks)	105	2014–2017
Everolimus (mTOR inhibitor)	NCT02008019	A phase II study of Everolimus in patients with primary or relapsed chondrosarcomas (CHONRAD)	II	2.5 and 10 mg/day for 30 days	Success rate per dose defined as a decrease of KI67 expression (> 10%)	57	(2014–2019) suspended due to the unavailability of Everolimus
AG-120 (mutant IDH1 inhibitor)	NCT02073994	Study of orally administered AG-120 in subjects with advanced solid tumors, including glioma, with an IDH1 mutation	I	AG-120 p.o. administered continuously as a single agent on days 1–28 of a 28-day cycle	Safety Maximum tolerated dose	170	2014–2017
AG-221 (mutant IDH1 inhibitor)	NCT02273739	Study of orally administered AG-221 in subjects with advanced solid tumors, including glioma, and with angioimmunoblastic T-cell lymphoma, with an IDH2 mutation	I/II	AG-221 p.o. administered every day of 28-day cycles until disease progression or unacceptable toxicities	Safety Maximum tolerated dose	21	2014–2017
Nivolumab (PARP inhibitor) + Ipilimumab (anti-CTLA4 antibody)	NCT02982486	A phase II of nivolumab plus ipilimumab in non-resectable sarcoma and endometrial carcinoma	II	Nivolumab 240 mg i.v. every 2 weeks plus Ipilimumab 1 mg/m^2^ i.v. every 6 weeks	Progression-free survival and therapeutic response evaluated by RECIST 1.1	60	2017–2020
Pembrolizumab (anti-PD1)	NCT02301039	SARC028: a phase II study of the anti-PD1 antibody pembrolizumab (MK-3475) in patients with advanced sarcomas	II	Pembrolizumab i.v. 200 mg every 3 weeks	Objective response rate evaluated according to RECIST 1.1	80	2015–2018

### Novelties in Osteosarcoma and Ewing Sarcoma

New therapeutic approaches have been proposed and are currently on-going to improve the survival rate of osteosarcoma patients [109, 112]. Similar strategies are now proposed for Ewing sarcoma patients.

### New Formulation of Chemotherapeutic Agents

In order to reduce its cardiotoxicity, liposomal doxorubicin formulations have been designed and show similar efficacy than conventional doxorubicin [117]. Liposomal doxorubicin is currently tested in phase I in refractory paediatric solid tumours (Table 2). Liposomal formulation can also be used for the modulation of drug pharmacology profiles such as irinotecan for which its pharmacology has likely limited its clinical activity. Positive benefit of irinotecan sucrosofate liposomes was demonstrated in a xenograft model of Ewing sarcoma and is assessed in a Phase trial (NCT02013336) [118].

### Tyrosine Kinase Inhibitors as Multiple Target Drugs

It is recognised that cytotoxic cancer agents can kill proliferating cells by damaging DNA or microtubules. Although numerous cancer cells are sensitive to chemotherapy despite their low proliferation, called «the proliferating rate paradox» by Mitchison TJ, quiescent cells are usually insensitive to cytotoxic agents [119] and can be reactivated in an adequate microenvironment [29, 84, 91]. In this context, the disruption of the dialogue between cancer cells and their microenvironment is a promising therapeutic approach in bone sarcomas. Migration, survival and proliferation are controlled by a complex internal cell machinery but also by several external factors such as cytokines or growth-activating tyrosine kinase receptors [120]. Several clinical trials are in progress to assess tyrosine kinase inhibitors which are considered as multi-target drugs (Tables 2, 3; Fig. 3). Regorafenib an oral multikinase inhibitor targeting angiogenic factors (VEGFR1-3, TIE2), oncogenic kinases (KIT, RET, RAF) and pazopanib inhibiting VEGFR, PDGFR and cKIT are going to be assessed in osteosarcoma (Fig. 3) [120, 121]. First therapeutic response has been described in three metastatic osteosarcoma [122] and Ewing sarcoma [123] patients treated with pazopanib. Similarly, regorafenib showed its antitumour activity in osteosarcoma in a phase I clinical trial [124], a phase II is in progress and will include 126 patients treated daily with oral 160 mg regorafenib. Erlotinib targeting the EGFR, cabozantinib blocking cMET and entrectinib, a selective inhibitor of TrkA, B and C, C-ros oncogene 1 and ALK are also in phase II in rare tumours including Ewing sarcoma (Table 2).

### Bone Targeting

Ewing sarcoma cells activate osteoclastogenesis followed by increased bone resorption and in this context the blockade of osteoclast activation by a bisphosphonate showed therapeutic benefit in a pre-clinical model of Ewing sarcoma [71]. A phase III clinical trial including more than 1150 patients treated with zoledronate is currently on-going with primary completion by March 2019. Radium-223 (^223^Ra) is an alpha-emitting radiopharmaceutical compound which showed calcimimetic properties and consequently has intrinsic calcified tissue-targeting properties. Based on these specificities, the bone matrix is the preferential site of biodistribution. The first clinical evidence of response to radium-223 in osteosarcoma has been published by Subbiah et al. who described a reduction of bone pain and bone-remodelling parameters after treatment [125]. A phase I/II clinical trial is on-going (NCT01833520, «Phase I Dose Escalation of Monthly Intravenous Ra-223 Dichloride in Osteosarcoma») to determine the maximum tolerated dose of radium-223 dichloride for treating osteosarcoma patients. Fifteen patients have been enrolled and were treated with a starting dose of radium-223 dichloride (50 kBq/kg i.v. over several minutes on day 1 of each 4-week cycle). The final completion will be at the end of 2018 [112].

### DNA Repair Targeting

Poly(ADP-ribose) polymerase 1 (PARP1) is a key protein involved in DNA repair especially in DNA repair of single-strand breaks. In 2012, Garnett et al. have reported a high sensitivity of Ewing sarcoma cells to PARP inhibitors [126]. Based on this interesting observation, a first phase II trial was set up in Ewing sarcoma. Unfortunately, the results revealed the absence of efficacy of olaparib as a single agent [127]. However, pre-clinical studies demonstrated promising benefit when combining PARP inhibitors with other targeting pathways (e.g. IGF1 inhibition, Trabectedin, temozolomide) and justified several phase I clinical trials (NCT01858168, NCT02044120) [128]. In 2015, Kovac et al. studied 31 osteosarcoma samples by exosome sequencing and showed for the first time recurrent mutation signatures of BRCA deficiency [31]. This observation could be an excellent argument to assess the therapeutic efficacy of PARP inhibitors in osteosarcoma and clinical trials are in discussion.

### Immunotherapies

The immune system plays a key role in cancer and immune cells recruited by cancer cells (e.g. lymphocytes, dendritic cells, macrophages) are responsible for a local immune tolerance and T lymphocytes infiltrating osteosarcoma tissues [110]. Programmed cell death ligand 1 (PDL-1) is a cell-surface protein that represses the cytotoxic CD8^+^ T-cell-mediated immune response. PDL-1 is frequently highly expressed by cancer cells and has become a strategic target in oncology [109, 129, 130]. PD-1 and PDL-1 have also been reported to be expressed by some osteosarcoma, Ewing sarcoma and giant cell tumours of bone as well as in soft-tissue sarcoma [131]. Shen et al. analysed the expression of PDL-1 in osteosarcoma samples and revealed its expression in a subset of osteosarcoma as well as a correlation between PDL-1 expression and T lymphocyte infiltration [132]. More recently, Sandara et al. demonstrated an increased PDL-1 expression and T-cell infiltration in metastatic high-grade osteosarcoma strengthening the clinical interest of PDL-1/PD-1 inhibition in osteosarcoma [133]. Paoluzzi et al. retrospectively analysed a cohort of 28 patients with relapsed metastatic/unresectable soft-tissue and bone sarcomas, who were treated with i.v. nivolumab (anti-PD1) 3 mg/kg every 2 weeks with or without pazopanib at 400–800 mg daily [134]. They observed three partial responses, nine stable disease and twelve patients had progression of disease. The authors concluded that a clinical benefit was observed in 50% of the evaluable patients. Based on these observations, the assessment of two anti-PD1 antibodies, pembrolizumab (NCT02301039) and nivolumab (NCT02304458) are in progress in osteosarcoma (Fig. 3). PD-1 inhibitor (nivolumab) is currently assessed in Ewing sarcoma in combination with an anti CTLA-4 antibody (Ipilimumab) (Table 2).

Preparation of immune cells such as dendritic cells, loaded T lymphocytes and NK (natural killer) cells are also in evaluation in phase I/II clinical trials in osteosarcoma (Fig. 3) and Ewing sarcoma (Table 2). The main goal of these studies is to lift the local immune tolerance and to reactivate the immune response against cancer cells. Thus, a pilot study (NCT02409576) in which activated haploidentical NK cells will be administered in 20 sarcoma patients is currently being carried out. The primary outcome will be clinical response (estimated primary completion date: end 2018).

Macrophage infiltration contributes to the control of osteosarcoma growth [105–107]. From this observation, several therapeutic strategies have been developed. One of the more “polemical” agents is Mifamurtide (L-MTP-PE), a synthetic analogue of a bacterial wall component able to activate macrophages resulting in improvement of overall survival by around 10% in combination with chemotherapy [113]. However, due to some controversy on the design of the study, its use is not universally admitted and a phase II/III clinical trial is on-going (NCT01459484). This trial will enrol more than 200 patients. Mifamurtide [2 mg/m^2^ twice a week for the first 3 months, then weekly for the next 6 months (total length of treatment 44 weeks)] will be added as post-surgery regimen in association with chemotherapy. Patients will be identified as good or bad responders according to the expression levels of P-glycoprotein. The estimated primary completion date is beginning of 2020.

### Fusion Protein Targeting

Ewing sarcoma are characterised by a t(11; 22) (q24; q12) translocation resulting in the *EWS/FLi1* fusion gene considered as a driver gene for the disease. New therapeutic approaches targeting *EWS/FLi1* gene or the corresponding protein have been set up (Table 2).

Based on the pre-clinical data on EWS–Fli1 silencing [13, 14], a phase I clinical trial has been designed for the treatment of Ewing sarcoma patients by a shRNA EWS/Fli1 type lipoplex (NCT02736565, Table 2). A dose escalation study of intravenous shRNA EWS/Fli1 type lipoplex (up to 0.156 mg/kg of DNA/single dose) will be carried out. The drug will be administered twice a week for 4 weeks for a total of eight infusions per cycle followed by 2 weeks of rest. Adverse effect and the therapeutic response will be assessed (estimated study completion date: end 2019). Similarly, TK216 is a chemical compound developed to inhibit downstream effects of the EWS–FLi1 transcription factor (NCT02657005, Table 2). The maximum tolerated dose will be determined in a phase I clinical trial.

### Cyclin-Dependent Kinase Inhibitors

CDK4 and CDK6 are kinases involved in the control of the cell cycle and act in G1 phase. In order to block cell proliferation, cyclin-dependent kinase inhibitors have been designed. Among them, Abemaciclib inhibits CD4 and CDK6 and induces a cell cycle arrest in G1 phase by acting on Rb phosphorylation. Fifty patients including osteosarcoma and Ewing sarcoma patients will be enrolled in a phase I clinical trial (NCT02644460) to determine the maximum tolerated dose (estimated study completion date: 2020).

### Disialoganglioside (GD2) Targeting and Drug Resistance (gpNMB)

A recent study revealed that most osteosarcoma and Ewing sarcoma expressed GD2, which is suspected to enhance tumour aggressiveness [135]. A phase II clinical study is in progress [NCT02502786, «Humanized monoclonal antibody 3F8 (Hu3F8) with Granulocyte-Macrophage Colony-Stimulating Factor (GM-CSF) in the treatment of recurrent osteosarcoma»]. Patients are treated with three doses of hu3F8 (2.4 mg/kg/dose for 3 days) and 10 days of GM-CSF (five cycles maximum). The primary outcome is the event-free survival. Glycoprotein non-metastatic melanoma protein B (gpNMB) is highly expressed in solid tumours and promotes metastatic progression by modulation of invasion and migration. A phase II clinical trial (NCT01353625) is evaluating the therapeutic benefit of Glembatumumab vedotin, an antibody-drug conjugate targeting gpNMB, in osteosarcoma patients. Patients with recurrent disease or refractory to conventional therapy have been included. The primary outcome is the dose-limiting toxicity and non-tolerated dose (estimated primary completion date: end 2018).

### Novelties in Chondrosarcoma

Chondrosarcoma comprises chemo- and radioresistant tumours with high risk of recurrence and surgery remains the treatment of choice. Due to their common origin, numerous new clinical approaches are similar to those proposed for osteosarcoma and Ewing sarcoma (Table 3). Because chondrosarcoma cells are sensitive to soluble factors produced by their microenvironment and activate various tyrosine kinase receptors, several tyrosine kinase inhibitors are clinically assessed alone or in combination: regorafenib, pazopanib, dasatinib (Bcr-Abl and Src family tyrosine kinase inhibitor), imatinib (Bcr-Abl, cKIT, RET, NGF-R, PDGFRα/β, ABL1, M-CSFR). mTOR plays a role in the control of numerous basic biological functions such as proliferation and migration and acts as a nutriment sensor. One of the best inhibitors used in clinic is rapamycin (sirolimus) which can inhibit mTOR after its binding to FKBP12 and acts as an immunosuppressive agent. Bernstein-Molho et al. analysed the effect of mTOR inhibition by sirolimus combined with cyclophosphamide in a series of 49 recurrent unresectable chondrosarcomas [136]. The combination of both agents was well tolerated with no significant adverse effects and could have therapeutic benefit. Indeed, 10% of objective response and 60% of stabilisation of disease for at least 6 months were observed. A phase II clinical trial is on-going associating both agents in unresectable chondrosarcoma (NCT02821507, Table 3). Everolimus, targeting mTORC1 (mTOR complex 1), appeared efficacious as single agent in a rat chondrosarcoma model [137] and a phase II clinical trial has been designed to evaluate its therapeutic efficacy in primary or relapsed chondrosarcoma (NCT02008019, Table 3).

IDH-1 or -2 are frequently mutated in malignant cartilaginous tumours and two phase I clinical trials are in progress with AG-120, a mutant IDH-1 inhibitor (NCT02073994) and AG-221, a mutant IDH2 inhibitor (NCT02273739).

Chondrosarcoma development is associated with the infiltration of immune cells [65]. In an in vivo rat chondrosarcoma model, Simard et al. demonstrated a positive impact on tumour growth after selective T cell depletion in contrast to the depletion of CD163^+^ macrophages resulting in a slowdown of tumour development [65]. These results showed the clear implication of the immune system on the pathogenesis of chondrosarcoma and the clinical interest to assess new inhibitors of immune checkpoints. These observations were confirmed more recently by Kostine et al. who demonstrated that 41–52% of dedifferentiated chondrosarcomas displayed PD-L1 positivity [138]. A phase II clinical trial is on-going and patients will be treated by intravenous pembrolizumab at 200 mg every 3 weeks (NCT02301039, Table 3).

## Giant Cell Tumours of Bone: Benign Tumours with Malignant-Like Properties

**Fig. 4:**
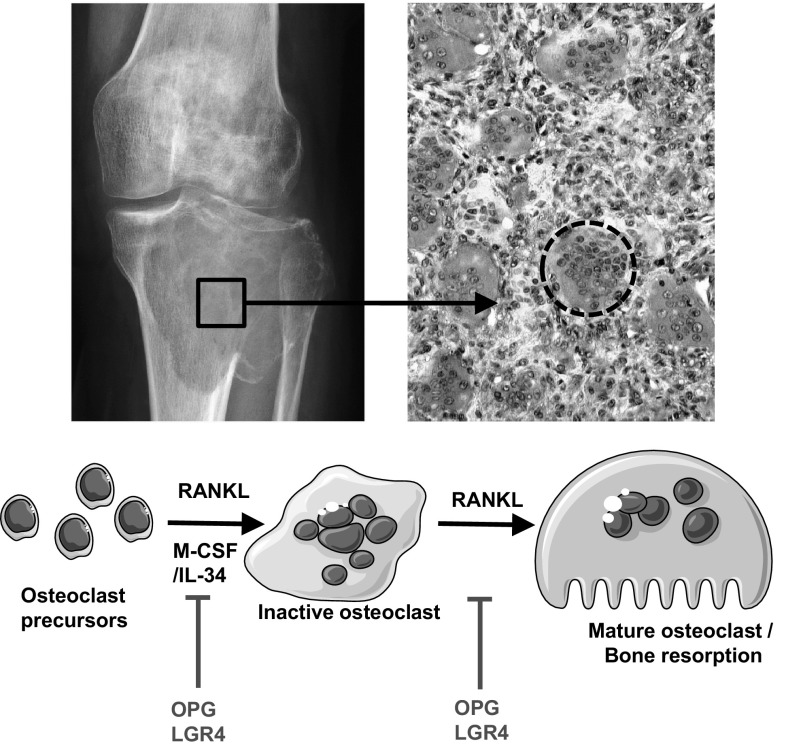
Giant cell tumours of bone: a benign entity with malignant features. Giant cell tumours of bone are composed of three main cell populations: stromal cells, macrophages and multinucleated osteoclast-like cells. These tumours are responsible for a marked local bone resorption leading to the formation of large osteolytic foci easily detectable by X-ray radiography. RANKL/M-CSF and/or RANKL/IL-34 released by stromal cell could induce the differentiation of macrophages considered as osteoclast precursors towards immature and mature osteoclasts resorbing bone. Soluble OPG and membrane LGR4 are two receptors that negatively control osteoclastogenesis. OPG acts as a decoy receptor to RANK resulting in blocked RANKL–RANK interactions. LGR4 is expressed by osteoclasts and binds to RANKL leading to Gαq/GS3K-β signalling and repression of the NFATc1 molecular pathway. *IL-34* Interleukin-34, *LGR-4* G-protein-coupled receptor 4, *M-CSF* Macrophage Colony-Stimulating Factor, *OPG* osteoprotegerin, *RANKL* Receptor of Nuclear factor kappaB Ligand

In the field of bone sarcoma, giant cell tumours (GCTs) have a special status. Indeed, GCTs are benign tumours with no nuclear cytologic aberration, intensively damaging the host bone and the cells can spread to the soft tissue in a similar manner to a malignant tumour [139–141]. Indeed, high-grade malignant neoplasm can be identified at the time of diagnosis or subsequent surgery (secondary malignancy in GCT) or radiotherapy. Giant cell tumours of bone are rare tumours with an incidence of around 1 new case per 100,000 people per year and affect mainly young adults on the second and third decade. The ratio male/female of 1:2 is in favour of the female. The tumour tissue is characterised by three main cellular components: (i) giant multinucleated cells (osteoclast-like cells), (ii) mononuclear macrophages and (iii) mononuclear stromal cells (Fig. 4). Stromal cells secrete numerous pro-myeloid factors such as M-CSF and pro-osteoclastic factors such as RANKL resulting in monocyte/macrophage proliferation and osteoclastogenesis. Indeed, osteoclast precursors have monocytic/macrophagic origin and can proliferate, fuse and differentiate in the presence of M-CSF and RANKL (Fig. 4). RANKL is mandatory for osteoclastogenesis. RANKL binds to three distinct receptors: (i) RANK: a transmembrane receptor expressed at the surface of osteoclasts and their precursors and is responsible for osteoclast differentiation; (ii) OPG: a soluble decoy receptor blocking the binding of RANKL to RANK and therefore considered as an anti-bone catabolic agent, (iii) LGR-4 expressed at the cell membrane of osteoclasts and which negatively regulates osteoclast differentiation (Fig. 4) [142]. The origin of giant cell tumours of bone has been controversial for a long time. Nowadays, it is widely accepted that the stromal component is “the tumoural” element of the tissue and its dysregulation leads to the recruitment, proliferation and differentiation of macrophages. The clinical consequence is massive local bone destruction (Fig. 4). The current treatment is based on a resection surgery but unfortunately frequent recurrences associated with a high morbidity are observed. This is followed by a possible malignant transformation with a metastatic profile after up to 20 years.

Similar to other bone sarcomas, the local microenvironment is crucial in the tumour development and the osteolytic process. In this context, anti-bone resorption agents have been assessed in clinical trials with great success [143, 144]. A phase II clinical trial (NCT01564121) has assessed zoledronic acid in 24 patients [144]. The patients were treated with extensive intralesional curettage followed by five courses of bisphosphonate. Unfortunately, even if short adjuvant treatments with zoledronic acid were associated with a low rate of recurrence, the study did not show any significant impact on local recurrence. Denosumab, a humanised blocking antibody against RANKL, is currently evaluated in a series of 586 patients in a phase II clinical trial (NCTNCT00680992) [102]. Denosumab was administered subcutaneously at a dose of 120 mg every 4 weeks and a loading dose of 120 mg s.c. on study days 8 and 15. The intermediate results showed the safety of the drug and first clinical benefit with at least 90% of tumour necrosis after denosumab administration (estimated completion date: end 2017). Preoperative pretreatment is currently in discussion to facilitate the surgical resection in patients with aggressive tumours with high-risk location (e.g. spine).

## Conclusion

Bone sarcomas are rare and heterogeneous diseases. Most bone sarcomas originate from MSCs and share a common feature with a marked implication of the local environment in their pathogenesis. This microenvironment appears as an impressive source of therapeutic targets and is leading to the design of numerous promising clinical trials. However, the tumour microenvironment of bone sarcomas is also very heterogeneous and includes numerous cell types, all of them composed by heterogeneous sub-clones. A better characterisation is the key challenge for a better patient stratification and development of personalised medicine.
